# Investigation on the I–V Kink Effect in Large Signal Modeling of AlGaN/GaN HEMTs

**DOI:** 10.3390/mi9110571

**Published:** 2018-11-05

**Authors:** Shuman Mao, Yuehang Xu

**Affiliations:** School of Electronic Science and Engineering (National Exemplary School of Microelectronics), University of Electronic Science and Technology of China, Chengdu 611731, China; 201711020135@std.uestc.edu.cn

**Keywords:** I–V kink effect, AlGaN/GaN HEMT, large signal performance

## Abstract

The effect brought by the I–V kink effect on large signal performance of AlGaN/GaN high electron mobility transistors (HEMTs) was investigated in this paper. An improved compact model was proposed to accurately characterize the I–V kink effect. The bias dependence of the I–V kink effect has also been taken into consideration. AlGaN/GaN HEMTs with different gate width were utilized to validate the proposed model. Built on the proposed model, the effect brought by the I–V kink effect on large signal performance has been studied. Results show that the I–V kink effect will lead to the degradation of characteristics, including output power, gain, and power-added efficiency at the saturation region. Furthermore, the influence of the I–V kink effect was found to be related with the input power and the static bias point in this work. The time domain waveform and AC dynamic load line were used for validation of results based on simulation. The consequences of this paper will be useful for the optimization of practical circuit design.

## 1. Introduction

Due to the special characteristics of the material itself, gallium nitride (GaN) has been widely used in wireless applications, THz band emerging devices, space industry, power electronics, and many other fields [1,2,3]. Also, with the development of fabrication techniques, the feature size of GaN-based devices have been shrinking to less than 100 nm [4]. Together with its unique characteristics, especially higher breakdown voltage and higher output power density, it has been proven to be an excellent candidate in high frequency applications [5]. Numerous circuit designs based on GaN processes with outstanding performance have sprung out these recent years [6,7,8]. The rapid development of GaN-based devices also stimulates the improvement of compact modeling, which serves as a key to practical circuit design [9].

In the past few years, lots of work has been focused on the characterization of electrothermal [10,11,12] and trapping effects [13,14,15] in compact modeling of GaN high electron mobility transistors (HEMTs). Many compact models, such as EE_HEMT1 model [16], Angelov GaN model [17], MVSG model [18], and ASM GaN model [19], have been developed for accurate characterization of device performance. Besides the development of the core model mentioned above, other real device effect models, such as noise model [20] and gate current model [19], are also proposed to improve the core model. I–V kink effect is also a common phenomenon observable in several kinds of transistors [21]. The mechanism has been thoroughly studied in many works. The setting of maximum value of drain-source voltage [22], as well as the sweeping direction of drain-source voltage in measurement [23], will also affect the kink degree. Also, the influence brought by the I–V kink effect on S parameters has been studied in [24]. However, the characterization of the I–V kink effect in compact model [25], and the influence brought by it on the large signal performance, have seldom been reported. As the I–V kink effect is observable when the device is biased at linear region, the load line will be influenced by it. In terms of practical circuit design, especially for high linearity and many other applications, it is worthy to develop an accurate I–V kink effect model and investigate the influence brought by it on large signal performance.

In this paper, the effect brought by the I–V kink effect on large signal performance of AlGaN/GaN HEMTs was studied. An improved compact model was proposed to accurately characterize the I–V kink effect. The model was validated via GaN HEMTs with different gate widths. Built on the proposed model, the effect brought by the I–V kink effect on large signal performance has been studied with different input power and under different bias points, separately. The time domain waveform and AC dynamic load line were also used for validation of results based on simulation in this work.

This paper is organized as follows. In Section 2, the model description was presented. The modeling method of the I–V kink effect was given in detail. In Section 3, the proposed model was validated by two AlGaN/GaN HEMTs with different gate width, at first. Then, the influence brought by the I–V kink effect with different input power and under different bias points was analyzed separately. Finally, in Section 4, the conclusion of this work is presented.

## 2. Model Description

### 2.1. Characterization of the I–V Kink Effect

**Figure 1:**
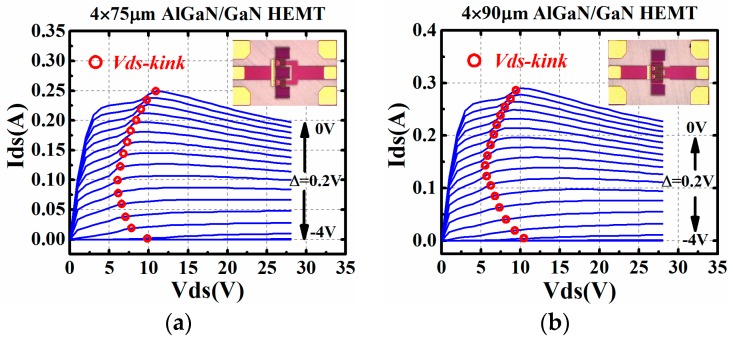
I–V kink effect in 0.25 μm AlGaN/GaN HEMTs with different gate width: (**a**) 4 × 75 μm and (**b**) 4 × 90 μm.

The I–V kink effect is a common physical phenomenon in several kinds of transistors. Trapping effects have been proved to be the main reason for the occurrence of I–V kink effect [26]. Due to the defect induced by fabrication, the trap distributed in devices will lead to the current collapse when devices are biased at linear region [27]. However, along with the increase of drain-source voltage, the rise of the electric field in the channel will assist the de-trapping process. This will, in the end, lead to the “jump” of drain-source current [28]. The drain-source voltage *V_ds_,* when the current recovers, is called *V_ds_kink_* in numerous works. This phenomenon can also be captured in the AlGaN/GaN HEMTs used in this work. I–V curves of GaN HEMTs with different gate width are presented in Figure 1. It is worthy to mention here that the AlGaN/GaN HEMTs used in this work were fabricated in WIN SEMICONDUCTORS Corporation NP25-00 Gallium Nitride process. These devices were grown on a 4 mil and 100 μm thickness SiC substrate.

As the I–V kink effect only leads to the current collapse when *V_ds_* is lower than 10 V in Figure 1, modification is needed to revise the nonlinear current formulation in the conventional compact model. In order to improve the convergence of the compact model, a simplified version based on the method in [25] was employed, in this work, to characterize the kink effect. The parameter *V_dsi_* in [25] was not found to be suitable in this work, and was replaced by drain-source voltage *V_ds_* to simplify the model. Also, as the parameter *V_ds_k_*_0_ in [25] directly determines the value of *V_ds_kink_* in Figure 1, it has been changed to *V_ds_kink_* in this work. The formulation of the whole I–V kink model is shown in Equation (1).

(1a) $$I_{kink}=I_{k}\times \left(1+\tanh\left(\frac{V_{ds}-V_{ds\_kink}}{V_{ds\_k1}}\right)\right),$$

(1b) $$I_{k}=I_{k0}\times \exp\left(-{\left(\frac{V_{gs}-V_{gs\_k0}}{V_{gs\_k1}}\right)}^{2}\right),\;$$

where *I_k_*_0_, *V_ds_k_*_1_, *V_ds_kink_*, *V_gs_k_*_0_, and *V_gs_k_*_1_ are parameters which can be achieved by measured I–V curve.

In Equation 1, *I_k_*_0_ denotes the maximum difference between the *I_ds_* curve with I–V kink effect, and the one without I–V kink effect [25]. The *V_gs_k_*_0_ refers to the gate-source voltage *V_gs_* when *I_k_* is equal to *I_k_*_0_. Since the GaN HEMT in this work is a kind of depletion mode device which is mainly for power amplifier application, gate-source voltage *V_gs_* is equal to or less than zero. As a result, *V_gs_k_*_0_ is equal to zero in this work. *V_gs_k_*_1_ is a parameter used for describing the variation of *I_k_* under different *V_gs_*. It can be achieved by fitting *I_k_* under different *V_gs_*. *V_ds_k_*_1_ denotes the slope of the I–V curve transferring from the kink region to the region without kink. *V_ds_kink_* refers to the drain-source voltage when the I–V kink effect disappears in the I–V curve.

However, with the model in Equation (1), the value of *V_ds_kink_* remains the same under each *V_gs_*. The bias dependence of *V_ds_kink_* cannot be accurately characterized only with Equation (1) because the parameter *V_ds_kink_* is not an expression related to the gate-source voltage *V_gs_*. As the variation of *V_ds_kink_*, along with the change of *V_gs_* shown in Figure 1, agrees well with the trend of a cubic function, the formulation in (2) was used in this work to add bias dependence into parameter *V_ds_kink_* in Equation (1).

(2) $$V_{ds\_kink}=aV_{gs}{}^{3}+bV_{gs}{}^{2}+cV_{gs}+d,$$

where *a*, *b*, *c*, and *d* are all fitting parameters*.* They can be achieved by polynomial fitting with the extracted discrete *V_ds_kink_* under different *V_gs_*, which have been marked in red circles in Figure 1.

With the parameter extraction method mentioned above, the I–V kink effect model can be achieved. The extracted parameters of the I–V kink effect model in Equations (1) and (2) for the 4 × 75 μm AlGaN/GaN HEMT in Figure 1a are presented in Table 1.

### 2.2. Compact Modeling and Its Validation

Comparing with other physical based [18,19] or empirical [16] compact model, the Angelov model [17] takes advantage of good convergence and much easier parameter extraction. As a result, an empirical compact modeling method [29] based on the Angelov theory was used in this work to model the drain-source current of the GaN HEMT. With the modification based on Equations (1) and (2), the bias dependence of the I–V kink effect can be accurately described. The self-heating effect was modeled by the variation of channel temperature and the trapping effect was modeled by the equivalent gate voltage method [15] in the compact model of this work. Then, the I–V kink effect model is integrated into the compact model by direct addition into the current model expression shown in Equation (3a). The proposed model in this work is also scalable. The capacitance model, including *C_gs_* and *C_gd_* mentioned in [29], is used in this work.

(3a) $$I_{ds}=I_{ds\_nkink}+I_{kink},$$

(3b) $$I_{ds\_nkink}=I_{pkth}\times \left(1+M_{ipkth}\times \tanh\left(\psi \right)\right)\times \tanh\left(\alpha V_{ds}\right),\;$$

where *I_pkth_*, *M_ipkth_*, *ψ*, and *α* are all model parameters of the improved Angelov model in [29]. *I_kink_* denotes the expression in Equation (1a).

**Figure 2:**
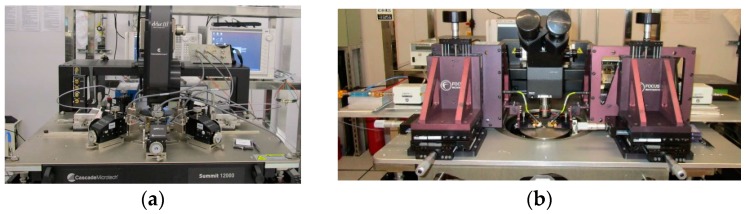
The on-wafer measurement system. (**a**) The measurement desk for DCIV and S parameter; (**b**) The on-wafer load-pull system.

The static DCIV curves in this work were measured on cascade deck (Summit 12000, FormFactor, Livermore, CA, USA) with the help of power device analyzer/curve tracer (Keysight B1505A, Keysight Technologies, Santa Rosa, CA, USA). The photography of the on-wafer measurement system is shown in Figure 2a.

The on-wafer load-pull system used for achieving maximum output power is shown in Figure 2b. The measurement is performed on Cascade Summit 11000 (FormFactor, Livermore, CA, USA). The testing block diagram is the same as the one mentioned in [30]. The main instruments in this work include the source and load tuner (Focus CCMT-5080, Focus Microwaves Inc., Québec City, QC, Canada), vector network analyzer (Agilent N5245A, Keysight Technologies, Santa Rosa, CA, USA), DC power (Agilent E3633A/E3634A, Keysight Technologies, Santa Rosa, CA, USA), and the input signal amplifier (Agilent 83020A, Keysight Technologies, Santa Rosa, CA, USA). S parameters in this work are measured with Agilent N5247 (Keysight Technologies, Santa Rosa, CA, USA), and the DCIV curves are measured with Keysight B1505A (Keysight Technologies, Santa Rosa, CA, USA).

**Figure 3:**
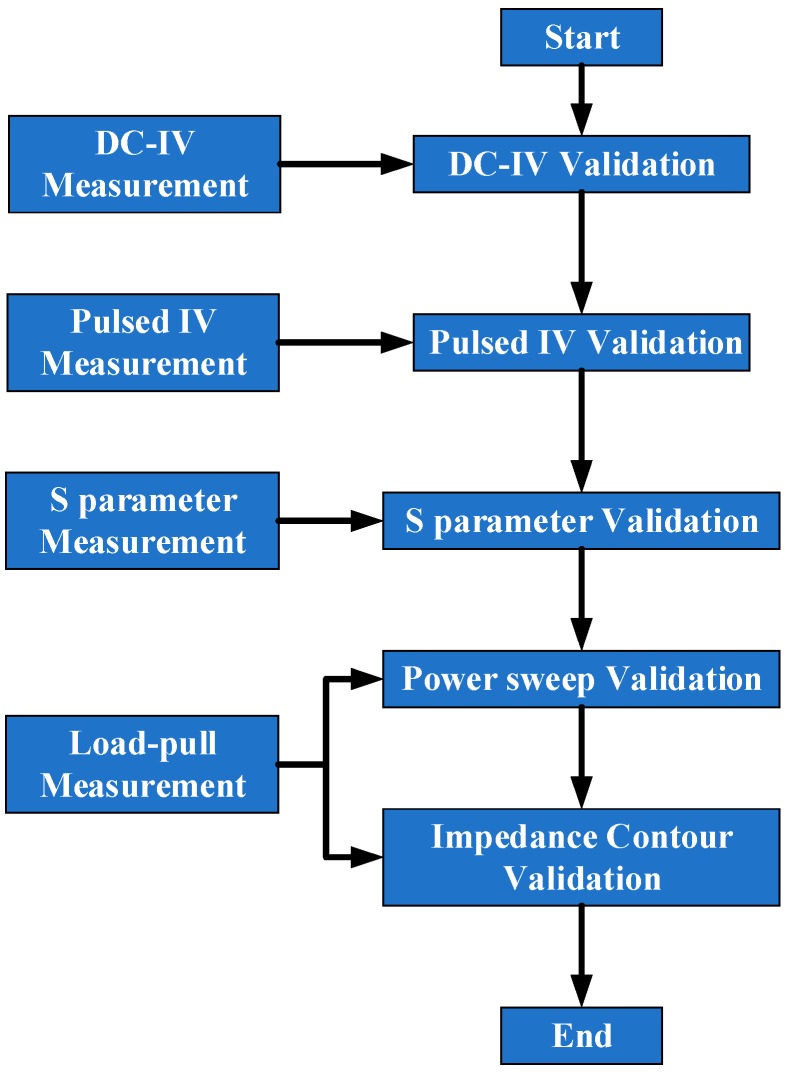
Flow chart of the validation procedure of the proposed model.

In order to validate the proposed model, two AlGaN/GaN HEMTs with the gate width of 4 × 75 μm and 4 × 90 μm were used. The flow chart for validation procedure is presented in Figure 3.

**Figure 4:**
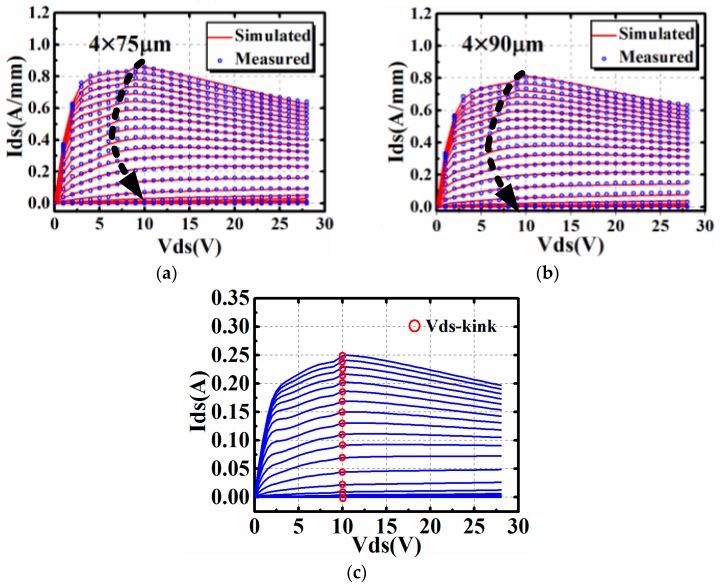
Validation of DCIV characteristics of the proposed model: (**a**) 4 × 75 μm GaN HEMT, (**b**) 4 × 90 μm GaN HEMT and (**c**) Results of the modeling approach in [25].

The comparison between simulation results and measured data of the DCIV of 4 × 75 μm and 4 × 90 μm GaN HEMTs at room temperature are shown in Figure 4. The gate-source voltage *V_gs_* is swept from −4 V to 0 V stepped by 0.2 V, and drain-source voltage *V_ds_* is from 0 V to 28 V, stepped by 1 V to include the I–V kink effect.

Comparing with Figure 4c, it is clear that the bias dependence of *V_ds-kink_* in Figure 4a,b can be accurately described under different gate-source voltage *V_gs_* based on the proposed bias dependence model in Equation (2).

## 3. Investigation on Large Signal Performance

### 3.1. Validation of the Large Signal Model

**Figure 5:**
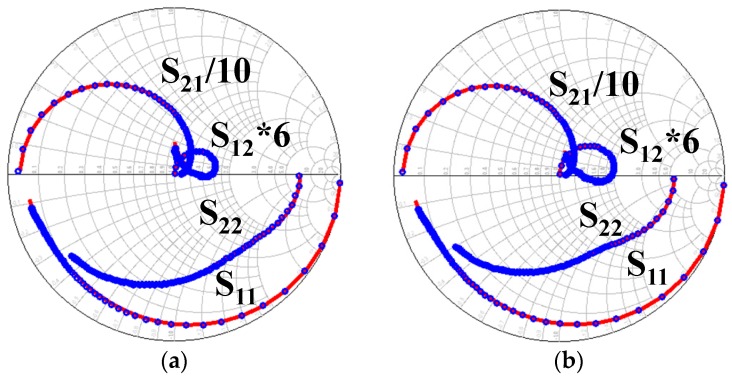
Small signal characteristic validation of the proposed model at different bias points: (**a**) *V_gs_* = −2.2 V, *V_ds_* = 20 V and (**b**) *V_gs_* = −2.6 V, *V_ds_* = 28 V.

The large signal model was embedded into Keysight ADS by symbolically defined device (SDD) tool. The small signal characteristics of the model have been validated at first. Results for S parameters at *V_gs_* = −2.2 V, *V_ds_* = 20 V and *V_gs_* = −2.6 V, *V_ds_* = 28 V are presented in Figure 5. The frequency band is from 0.1 GHz to 40 GHz in Figure 5.

**Figure 6:**
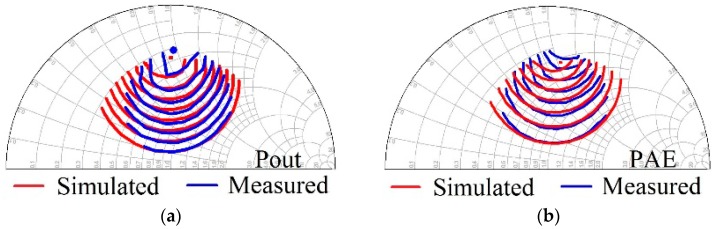
Validation of the impedance contours of the proposed model: (**a**) Impedance contour for maximum output power and (**b**) Impedance contour for maximum PAE.

It can be observed in Figure 5 that the proposed model can accurately characterize the small signal characteristics under different bias points over the frequency band. Then, in order to validate the large signal characteristics of the model, on-wafer load-pull measurement was performed to achieve the impedance of maximum output power. The impedance contours of a 4 × 75 μm GaN HEMT for maximum output power and maximum power-added efficiency (PAE) are presented in Figure 6.

**Figure 7:**
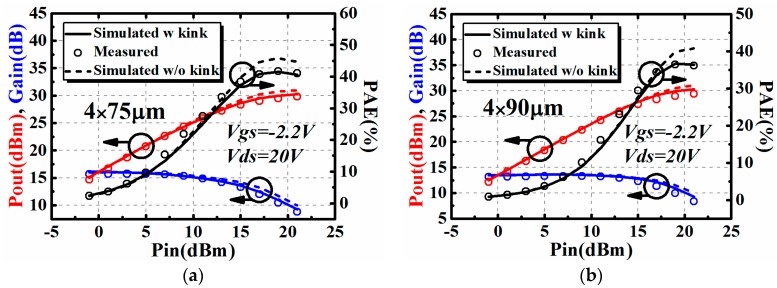
The influence brought by the I–V kink effect on large signal performance of AlGaN/GaN HEMTs @10GHz: (**a**) 4 × 75 μm and (**b**) 4 × 90 μm.

It is clear in Figure 6 that the proposed model can accurately predict the impedance contours. Terminated at the optimum source and load impedance, power sweep characteristics at a certain bias and frequency point can be achieved. The power sweep characteristics, including output power (Pout), gain, and power-added efficiency (PAE), are also validated for the AlGaN/GaN HEMTs with gate width of 4 × 75 μm and 4 × 90 μm. Results when the frequency of the input signal is 10 GHz are presented in Figure 7.

The influence brought by the I–V kink effect on large signal performance was also studied in Figure 7. The comparison between two conditions when one has taken the I–V kink effect into consideration, while the other has not, are shown in Figure 7. It can be seen in Figure 7 that the I–V kink effect will lead to the degradation of large signal performance, including output power (Pout), gain, and power-added efficiency (PAE) at the saturation region. In terms of output power and gain, the degradation induced by the I–V kink effect is only 1 dB or even smaller. This degradation is in an acceptable range for circuit design. However, the degradation of power-added efficiency is observable, comparing with output power and gain. This should be taken into consideration in practical circuit design, especially high efficiency applications.

**Figure 8:**
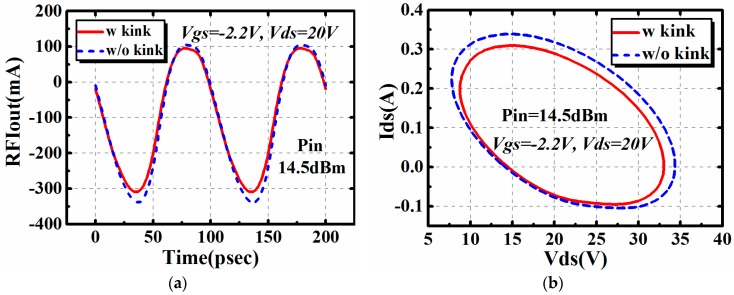
The influence brought by the I–V kink effect on time domain characteristics of AlGaN/GaN HEMTs @10GHz: (**a**) time domain waveform and (**b**) AC dynamic load line.

Besides the large signal performance at a certain frequency point, the influence brought by the I–V kink effect on time domain characteristics was then studied by simulation. The 4 × 75 μm AlGaN/GaN HEMT was used for investigation. It was terminated in its optimum impedance for maximum output power at 10 GHz. The input impedance *Z_s_* is 14.05 + j * 18.50 Ω, while the output impedance *Z_L_* is 30.37 + j * 48.27 Ω. The input power was set to 14.5 dBm. Results are shown in Figure 8.

It is clear in Figure 8 that the I–V kink effect affects both the time domain waveform and the AC dynamic load line. Also, the I–V kink effect only affects the magnitude of output waveform. The phase remains the same in two conditions.

### 3.2. The Influence of I–V Kink Effect with Different Input Power

**Figure 9:**
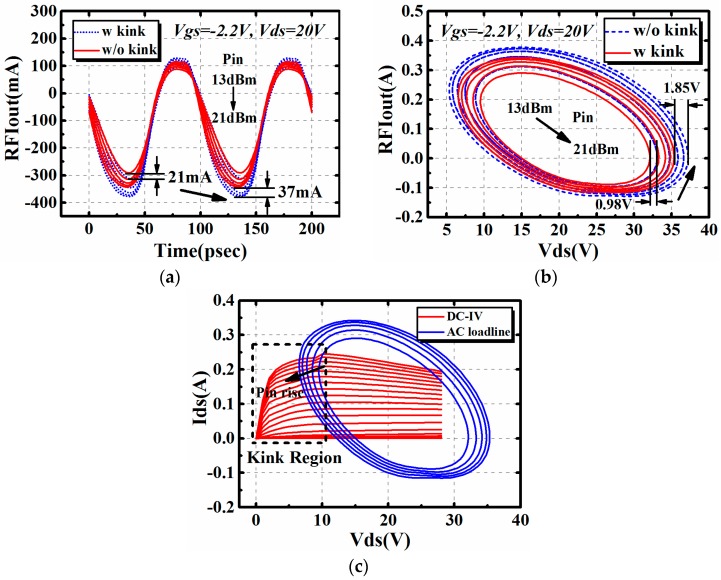
Investigation on influence brought by the I–V kink effect on time domain characteristics with different input power @10 GHz: (**a**) time domain waveform, (**b**) AC dynamic load line and (**c**) DCIV and AC dynamic load line.

In order to further validate the influence brought by the I–V kink effect on large signal performance shown in Figure 7 on the aspect of input power, the time domain characteristics of the 4 × 75 μm AlGaN/GaN HEMT used in this work were studied. Characteristics, including the AC dynamic load line and output time domain waveform of the device with different input power, are shown in Figure 9. The input power was swept from 13 dBm to 21 dBm for investigation of different working states of the 4 × 75 μm GaN HEMT.

It can be observed in Figure 9a,b that, with the increase of input power, the influence brought by the I–V kink effect aggravates for both time domain waveform and AC dynamic load line. The decrease of the amplitude of the waveform will, in the end, lead to the degradation of output performance presented in Figure 7. The decrease of the amplitude of the waveform can be further explained by the distribution of AC dynamic load lines with different input power, shown in Figure 9c. Along with the rise of input power, more parts of the trace of AC dynamic load line will be located in the kink region marked in the dashed box in Figure 9c. Nonlinear effects will be induced by the current collapse in the region at this time. As a result, the I–V kink effect-induced current collapse should be considered the main reason for the variation shown in Figure 9a,b. Based on the analysis above, a compromising input power should be chosen in circuit design if the I–V kink effect is observable in the transistors.

### 3.3. The Influence of I–V Kink Effect under Different Bias Points

**Figure 10:**
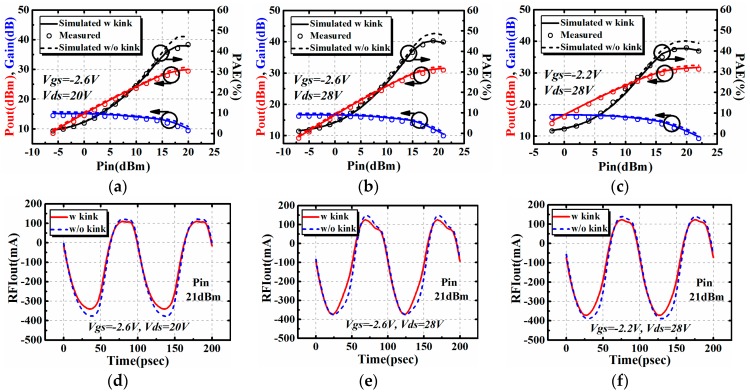
Investigation on the influence brought by the I–V kink effect at different bias points: (**a**–**c**) refers to the large signal output performance while (**d**–**f**) refers to the time domain waveform.

As the static bias point of a certain device also affects the distribution of AC dynamic load line, the bias dependence of the influence brought by the I–V kink effect has also been studied. The large signal performance at bias points, including *V_gs_* = −2.6 V, *V_ds_* = 20 V, *V_gs_* = −2.6 V, *V_ds_* = 28 V, *V_gs_* = −2.2 V, and *V_ds_* = 28 V were investigated in Figure 10. The time domain waveforms for each bias point are also included. The input power chosen for investigation is 21 dBm because the device is saturated. The influence brought by the I–V kink effect is observable under this circumstance.

The influence brought by the I–V kink effect on the amplitude of the waveform weakens along with the variation of bias points from Figure 10a–c. The variation trend in Figure 10 is the same as the one in Figure 7, that there is degradation of characteristics, including output power, gain, and power-added efficiency at the saturation region. Also, the degradation of PAE is observable compared with output power and gain. As the influence of the I–V kink effect on power-added efficiency (PAE) is observable, the influence has been further calculated by *∆PAE* = *PAEnk* − *PAEk*. Where *PAEnk* refers to PAE calculated without the I–V kink effect, while *PAEk* refers to PAE calculated with the I–V kink effect. Results for four different bias points are listed in Table 2.

**Figure 11:**
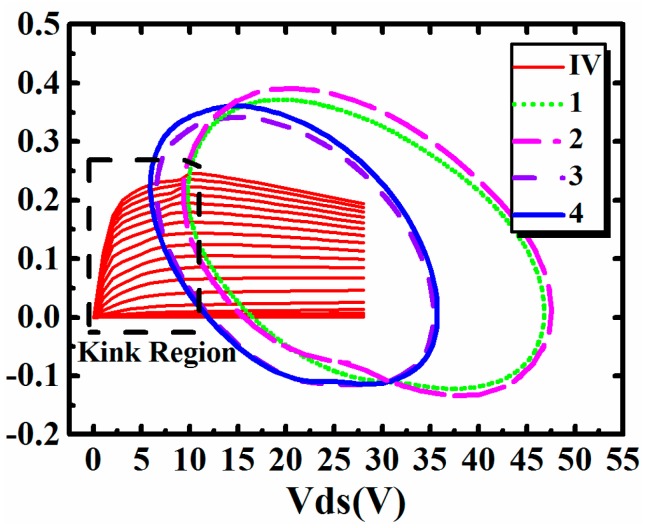
The distribution of AC dynamic load lines at four different bias points.

Table 2 shows that the influence of the I–V kink effect on PAE is related with both gate bias and drain bias. This phenomenon can be further validated by the distribution of AC dynamic load lines at these bias points. The input power was also 21 dBm, which was the same as the one in Figure 10. Results are shown in Figure 11. The index 1, 2, 3, and 4 in Figure 11 refer to the four bias points, which are consistent with the ones in Table 2.

It can be observed in Figure 11 that, with the rise of index presented in Table 2, more parts of each trace will be located in the kink region marked in the dashed box. As a result, the influence brought by the I–V kink effect will aggravate, at the same time. It can be concluded that the bias dependence of the influence is directly dependent on the length of the trace located in the kink region.

## 4. Conclusions

In this paper, the effect brought by the I–V kink effect on large signal performance of AlGaN/GaN HEMTs was investigated. An improved compact model was proposed to accurately characterize the I–V kink effect. The effect brought by the I–V kink effect on large signal performance has been studied with different input powers and under different bias points. Results show that the I–V kink effect will lead to the degradation of characteristics, including output power, gain, and power-added efficiency at saturation region. The degradation of output power and gain is in an acceptable range, while the degradation of PAE should be taken into consideration in circuit design. Results of this paper will be useful for optimization of practical circuit design.

## Figures and Tables

**Table 1:** The extracted I–V kink effect model parameters in Equations (1) and (2).

*I_k_*_0_	*v_ds_k_*_1_	*a*	*b*	*v_gs_k_*_0_	*v_gs_k_*_1_	*c*	*d*
0.04	0.012	−0.7	−1.01	0	3.08	2.51	9.82

**Table 2:** The influence of I–V kink effect on power-added efficiency.

Index	Bias Point	*∆PAE*
1	*V_gs_* = −2.2 V, *V_ds_* = 28 V	3.3%
2	*V_gs_* = −2.6 V, *V_ds_* = 28 V	3.8%
3	*V_gs_* = −2.2 V, *V_ds_* = 20 V	4.3%
4	*V_gs_* = −2.6 V, *V_ds_* = 20 V	5.1%
